# Characterization of Hsp17, a Novel Small Heat Shock Protein, in Sphingomonas melonis TY under Heat Stress

**DOI:** 10.1128/spectrum.01360-23

**Published:** 2023-07-12

**Authors:** Yihan Wang, Xiaoyu Wang, Hao Wu, Lvjing Wang, Haixia Wang, Zhenmei Lu

**Affiliations:** a MOE Laboratory of Biosystem Homeostasis and Protection, College of Life Sciences, Zhejiang University, Hangzhou, China; b Cancer Center, Zhejiang University, Hangzhou, China; State Key Laboratory of Microbial Resources, Institute of Microbiology, Chinese Academy of Sciences

**Keywords:** heat shock, small heat shock protein, coexpression analysis, cell morphology, stress resistance

## Abstract

Bacteria are constantly exposed to a variety of environmental stresses. Temperature is considered one of the most important environmental factors affecting microbial growth and survival. As ubiquitous environmental microorganisms, *Sphingomonas* species play essential roles in the biodegradation of organic contaminants, plant protection, and environmental remediation. Understanding the mechanism by which they respond to heat shock will help further improve cell resistance by applying synthetic biological strategies. Here, we assessed the transcriptomic and proteomic responses of Sphingomonas melonis TY to heat shock and found that stressful conditions caused significant changes in functional genes related to protein synthesis at the transcriptional level. The most notable changes observed were increases in the transcription (1,857-fold) and protein expression (11-fold) of Hsp17, which belongs to the small heat shock protein family, and the function of Hsp17 in heat stress was further investigated in this study. We found that the deletion of *hsp17* reduced the capacity of the cells to tolerate high temperatures, whereas the overexpression of *hsp17* significantly enhanced the ability of the cells to withstand high temperatures. Moreover, the heterologous expression of *hsp17* in Escherichia coli DH5α conferred to the bacterium the ability to resist heat stress. Interestingly, its cells were elongated and formed connected cells following the increase in temperature, while *hsp17* overexpression restored their normal morphology under high temperature. In general, these results indicate that the novel small heat shock protein Hsp17 greatly contributes to maintaining cell viability and morphology under stress conditions.

**IMPORTANCE** Temperature is generally considered the most important factor affecting metabolic functions and the survival of microbes. As molecular chaperones, small heat shock proteins can prevent damaged protein aggregation during abiotic stress, especially heat stress. *Sphingomonas* species are widely distributed in nature, and they can frequently be found in various extreme environments. However, the role of small heat shock proteins in *Sphingomonas* under high-temperature stress has not been elucidated. This study greatly enhances our understanding of a novel identified protein, Hsp17, in *S. melonis* TY in terms of its ability to resist heat stress and maintain cell morphology under high temperature, leading to a broader understanding of how microbes adapt to environmental extremes. Furthermore, our study will provide potential heat resistance elements for further enhancing cellular resistance as well as the synthetic biological applications of *Sphingomonas*.

## INTRODUCTION

As single-cell organisms, bacteria living in a fluctuating environment are constantly exposed to a variety of stress factors (1). The ability to sense and respond to changes in the environment is crucial for the adaptation and propagation of bacteria (2). Temperature has been proposed to have a direct impact on microbial survival (3). A sudden increase in temperature can destroy cell structures and even interfere with physiological functions (4, 5). Bacteria have evolved a variety of mechanisms to respond to high temperatures, which minimize damage and ensure the protection of cellular homeostasis. Heat stress or heat shock, resulting from exposure to temperatures over the physiological optimum, causes a change in gene expression in cells (6), resulting in deleterious effects on cells, including retarded growth and even death (7, 8). The heat shock response is a fundamental and critical cellular defensive mechanism against heat stress that results in the rapid expression of a group of proteins known as heat shock proteins (HSPs) (9). HSPs are chaperone molecules that participate in housekeeping functions such as folding newly synthesized polypeptides, refolding metastable proteins, assembling protein complexes, degrading misfolded proteins, and dissociating protein aggregates (10).

Small heat shock proteins (sHSPs), a family of HSPs, are characterized by a low subunit molecular mass of 12 to 43 kDa (10). As one of the most abundant groups of molecular chaperones in nature, sHSPs are widely found in various animals, plants, and microorganisms (11). Under stress conditions, sHSPs are rapidly upregulated to prevent protein aggregation (12). sHSPs differ from other heat shock protein families in that sHSPs themselves cannot refold unnatural proteins. They maintain protein homeostasis by binding substrate proteins in nonnative conformations, preventing irreversible aggregation, and then refolding, mediated by other ATP-dependent molecular chaperones (13). Under high temperatures, sHSPs have often been reported to prevent aggregation and assist in the folding of numerous proteins, resulting in balanced protein homeostasis in general. Furthermore, they have important functions in stress tolerance, membrane stability, and bilayer stability and assist in appropriate cell division in both prokaryotes and eukaryotes (14, 15). However, the expression of stress-responsive proteins, especially sHSPs, is affected by certain environmental factors, and their functions vary in different species.

*Sphingomonas* is a genus of Gram-negative bacteria that includes more than 103 species (16) that have been isolated from a wide range of habitats. They can also successfully thrive in a variety of extreme environments, such as volcanic lakes (17), thermal power plants (18), and high-temperature fermentation cellars (19). *Sphingomonas* has evolved an effective mechanism for resisting abiotic stresses, especially osmotic stress (20), and shows large changes in protein and RNA synthesis when faced with stress (21). *Sphingomonas* bacteria can also degrade numerous organic contaminants (22). Moreover, *Sphingomonas* species confer resistance to other bacterial pathogens in plants and affect plant secondary metabolism (23, 24). These benefits, however, would be diminished if these bacteria could not endure fluctuating ambient temperatures. Nevertheless, the biological functions of sHSPs in *Sphingomonas* have not been reported. Hence, analyzing the heat resistance mechanism of Sphingomonas melonis TY will reveal how microbes adapt to environmental extremes and increase our understanding of microbial ecology and evolution.

In this study, transcriptomic and proteomic coexpression analysis was performed to reveal the response of *S. melonis* TY to heat shock. Heat stress affects multiple cell processes, suppressing protein synthesis at the transcriptional level in particular. We therefore hypothesized that a unique heat resistance element that may be critical for protecting cells during heat stress conditions exists in *S. melonis* TY. Accordingly, a novel small heat shock protein, Hsp17, with a conserved α-crystallin structural domain was identified and characterized. Gene knockout and overexpression were performed to study the ability of *S. melonis* TY cells to resist stress and maintain normal cell division under high temperatures, and the function of Hsp17 in *S. melonis* TY was revealed.

## RESULTS

### Transcriptomic and proteomic analysis of *S. melonis* TY under heat shock.

**FIG 1:**
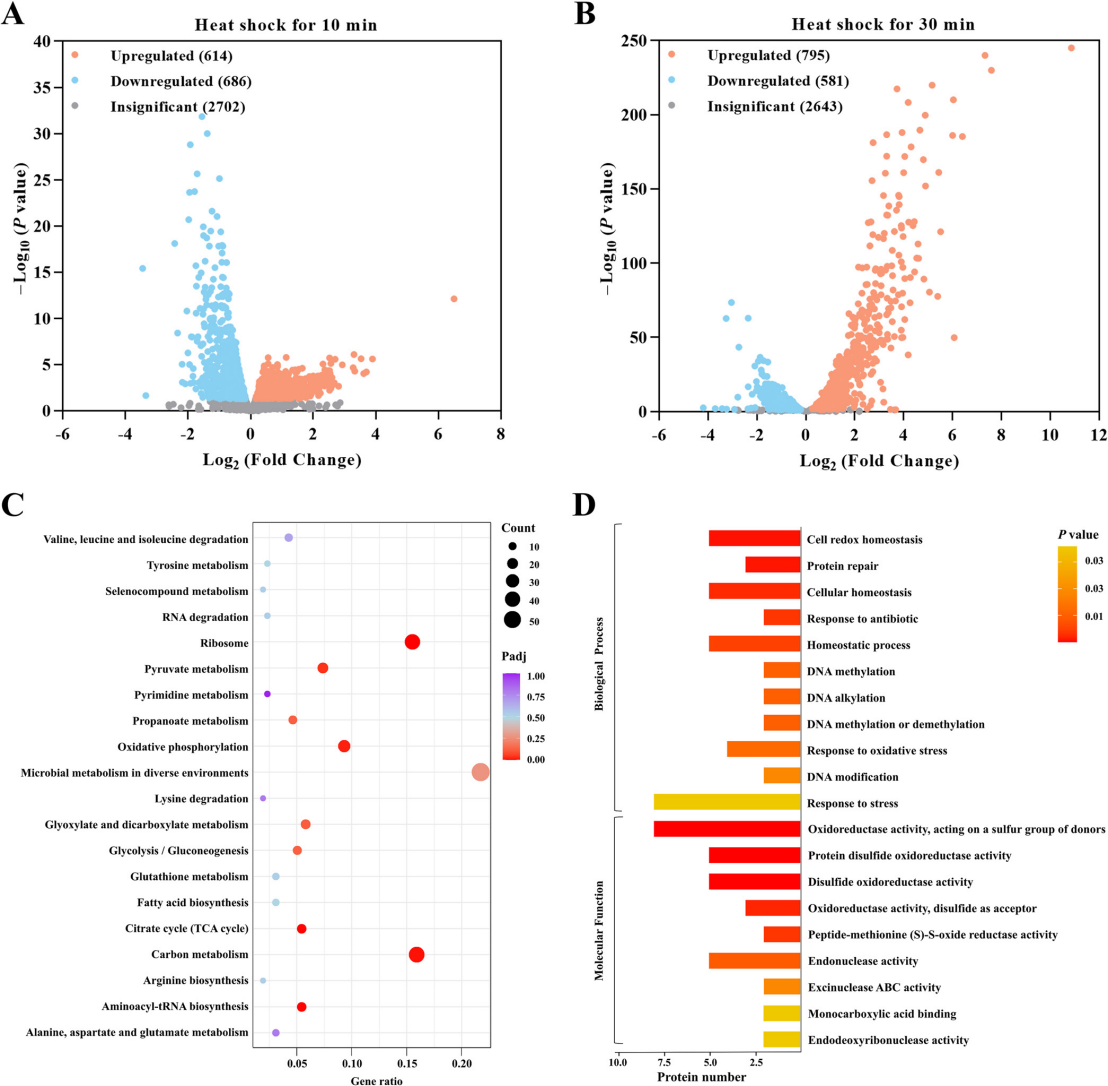
Gene and protein expression landscape of *S. melonis* TY under heat shock. Volcano plot of DEGs with a |log_2_ (fold change)| of >0 and an adjusted *P* value (padj) of <0.05 after 10 min (A) and 30 min (B) of heat shock. Orange dots represent upregulated genes. Gray dots represent genes with no significant expression change. Blue dots represent downregulated genes. (C) Functional classification of DEGs by KEGG pathway analysis using the KEGG BRITE database (top 20). (D) GO enrichment analysis of DEPs (top 20).

The transcriptomes of cells subjected to heat shock and cells without heat shock were analyzed and compared to identify the key factors involved in the response to heat. An obvious difference was observed between the two groups, indicating that transcripts were significantly altered in response to heat stress. After heat shock, the impact of time (75.27%) outweighed the impact of temperature (19.31%) (see Fig. S1 in the supplemental material). A total of 614 genes were upregulated, and 686 were downregulated after only 10 min of heat shock (Fig. 1A). In addition, 1,376 genes were differentially expressed after 30 min of heat shock; of these, 795 were upregulated and 581 were downregulated (Fig. 1B). Within 10 to 30 min of heat shock, the number of differentially upregulated genes increased by 29.48%, while the number of downregulated genes decreased by 18.07%. Venn diagram analysis of differentially expressed genes (DEGs) after 10 min and 30 min of heat shock revealed that 771 genes were differentially expressed at both time points, which was approximately 18.72% of all genes (Fig. S2A). Only these genes were considered in further transcriptomic analysis. Among all upregulated genes, the transcript level of BJP26_07370, which was annotated as an Hsp20/α-crystallin family member, was 89-fold and 1,856-fold higher after 10 min and 30 min of heat shock, respectively, than that after non-heat shock treatment (Table 1). According to Gene Ontology (GO) classification, DEGs related to the organonitrogen compound metabolism process, cell part, and ion binding terms were predominant in the biological process category (Fig. S2B). Additionally, KEGG analysis revealed that genes related to ribosomes, carbon metabolism, aminoacyl-tRNA biosynthesis, and oxidative phosphorylation were downregulated (Fig. 1C; Fig. S3). The largest number of DEGs was observed in the pathway of microbial metabolism in diverse environments (56 DEGs).

**TABLE 1:** Representative upregulated genes (top 10) in heat-shocked TY cells compared with those in non-heat-shocked TY cells^*a*^

Gene ID	FC 1	FC 2	Annotation
BJP26_07370	90.38	1,856.98	Hsp20/α-crystallin family
BJP26_00880	14.87	193.04	Calcium-binding protein
BJP26_02285	6.39	161.11	Chaperone protein ClpB
BJP26_00840	12.24	64.15	Hsp70 protein
BJP26_16085	5.06	84.89	Alkyl hydroperoxide reductase C
BJP26_05475	4.48	67.14	Cochaperonin GroES
BJP26_08430	9.67	29.84	RNA-binding protein Hfq
BJP26_05480	5.74	45.65	Chaperonin GroEL
BJP26_11430	3.80	43.41	Bacterial regulatory protein, ArsR family
BJP26_00700	7.01	23.10	Hypothetical protein

a FC 1, fold change after 10 min of heat shock; FC 2, fold change after 30 min of heat shock.

To further explore the effect of heat shock on *S. melonis* TY, we performed comparative iTRAQ proteomics to monitor dynamic protein changes. Ultimately, 31,419 peptides, 28,443 (90.53%) unique peptides, and 2,951 proteins were identified. Among these peptides, 171 were identified as differentially expressed proteins (DEPs), including 102 that were upregulated and 69 that were downregulated after 30 min of heat shock (Fig. S2C; Table 2). Among the proteins with upregulated expression levels, the protein WP_017978620.1 (BJP26_07370) showed an 11.24-fold increase after heat shock. The obtained information was then used to separate the DEPs into functional groups, and the results indicated that the DEP screen was representative of the effect of biological treatments on the samples (Fig. S2D). Further analysis of the GO-annotated proteins revealed that DEPs showed annotations only in the biological process and molecular function categories (Fig. 1D). Proteins related to oxidoreductase activity, acting on a sulfur group of donors, cell redox homeostasis, and protein repair were activated during heat shock. However, KEGG pathway annotation indicated that only the lipoarabinomannan biosynthesis pathway was significantly activated (Fig. S2E).

**TABLE 2:** Representative upregulated proteins (top 10) in heat-shocked TY cells compared with those in non-heat-shocked TY cells

Accession no.	FC^*a*^	Description
WP_017978620.1	11.24	Multispecies: Hsp20 family protein
WP_018250629.1	5.46	Hypothetical protein
WP_017980371.1	2.51	Hypothetical protein
WP_017977679.1	2.46	Multispecies: hypothetical protein
WP_017980667.1	2.02	AbrB/MazE/SpoVT family DNA-binding domain-containing protein
WP_197462282.1	2.00	Type IV secretory system conjugative DNA transfer family protein
WP_062125797.1	1.79	Hypothetical protein
WP_017979404.1	1.76	Multispecies: cold-shock protein
WP_017978092.1	1.71	Hypothetical protein
WP_017980346.1	1.68	ATP-dependent chaperone ClpB

a FC, fold change after 30 min of heat shock.

### Combined transcriptomic and proteomic analysis identified a novel small heat shock protein, Hsp17.

**FIG 2:**
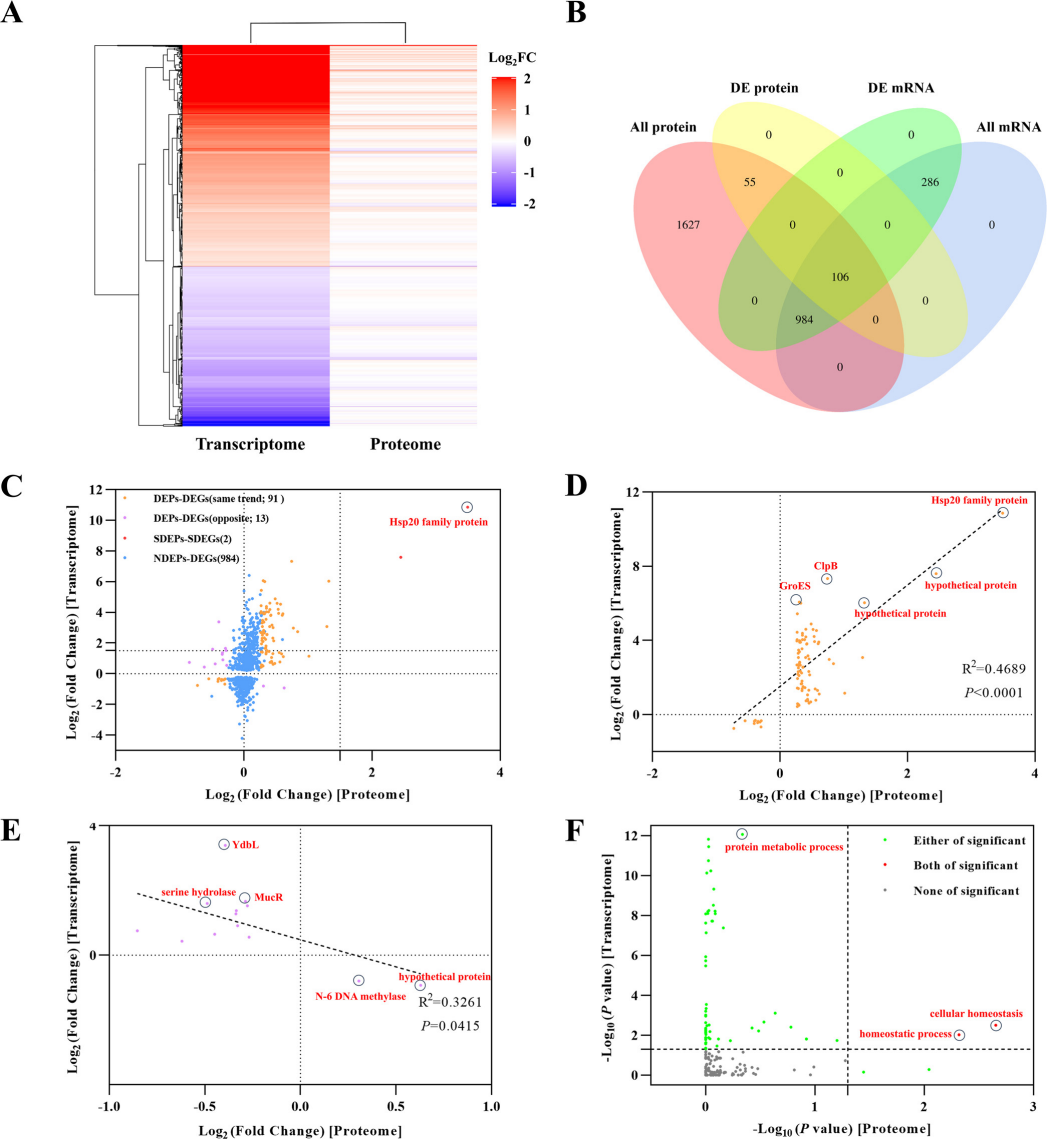
Transcriptomic and proteomic coexpression analysis of *S. melonis* TY after 30 min of heat shock. (A) Heat map of differentially expressed RNAs and proteins after 30 min of heat shock. (B) Venn diagram depicting the overlap and distribution of differentially expressed RNAs and DEPs after 30 min of heat shock. (C) Correlation of DEGs and DEPs. The density plot relates the proteomic log_2_ (fold change) with the transcriptomic log_2_ (fold change). The orange points represent the genes with significant expression showing the same trend in the proteome and transcriptome [DEPs-DEGs(same trend)], the purple points represent the genes with significant expression showing the opposite trend in the proteome and transcriptome [DEPs-DEGs(opposite)], the red points represent the genes expressed at super high levels according to both omics analyses (SDEPs-SDEGs), and the blue points represent the genes showing significant expression in the transcriptome but not in the proteome (NDEPs-DEGs). Dots within circles represent the typical differentially expressed proteins, same as below. (D) Correlation of genes with significant expression showing the same trend in the proteome and transcriptome. The linear fit is shown with a black dashed line. (E) Correlation of genes with significant expression showing opposite trends in the proteome and transcriptome. (F) Enrichment correlation analysis of GO categories. The green points represent the categories that were significantly enriched in either the proteome or the transcriptome, the red points represent the categories that were significantly enriched in both the proteome and transcriptome, and the gray points represent the categories that were not significantly enriched in either the proteome or the transcriptome.

A heat map was generated to illustrate the differential expression profiles of the identified transcripts and proteins (Fig. 2A). Most changes in gene expression induced by heat shock occurred at the transcriptional level. In total, 1,090 genes were annotated in both the transcriptome and proteome after 30 min of heat shock, of which 984 genes were regulated exclusively at the transcriptional level and were not enriched at the translational level and only 106 genes were differentially expressed at both levels, representing only 3.59% of the total proteins (Fig. 2B). There was a poor positive correlation between the changes in mRNA and protein, indicating that the effects of heat shock on protein abundance and transcript abundance were not exerted simultaneously. The vast majority of DEGs identified during heat shock were not associated with corresponding changes in protein expression. A scatterplot comparing the levels of each transcript (*y* axis) with the levels of its corresponding protein (*x* axis) showed a small heat shock protein, BJP26_07370 (WP_017978620.1), localized in the third quadrant (Fig. 2C), whose increase in expression exceeded the changes observed for any protein according to the proteomic data. In fact, the BJP26_07370 transcript and protein both accumulated at high levels in response to heat shock.

Among the above-mentioned 106 proteins, 93 showed the same expression trend (Fig. 2C, orange points), while only 13 presented the opposite expression trend (purple points). Proteins including the ATP-dependent chaperone ClpB, cochaperone GroES, and molecular chaperone DnaK were upregulated at both the mRNA and protein levels (Fig. 2D). However, proteins including YdbL family protein, MucR family transcriptional regulator, and serine hydrolase showed completely opposite expression trends, with upregulated expression at the transcript level and downregulated expression at the protein level, while N-6 DNA methylase showed the opposite expression trend (Fig. 2E). However, glutaredoxin and thioredoxin, which are involved in cellular homeostasis and homeostatic process, were differentially expressed in both the transcriptome and proteome data (Fig. 2F; Table S1). Not surprisingly, antioxidant enzyme systems and antioxidant nonenzymatic systems were activated in cells immediately after heat shock to defend against the stressful environment. Transcriptome and proteome coexpression analysis showed that only ribosome-associated pathways were differentially expressed at the transcriptional level, and no difference in total protein levels was observed (Fig. S4).

In combination with the above analysis, the BJP26_07370 gene product was characterized as a small heat shock protein with a molecular weight of 17.2 kDa, and as a result, it was given the name Hsp17. Hsp17 was expressed at extremely high levels according to both omics analyses, implying that it may play a vital role in the *S. melonis* TY response to heat stress. Therefore, it was selected for subsequent study.

### Characteristic description of the small heat shock protein Hsp17.

**FIG 3:**
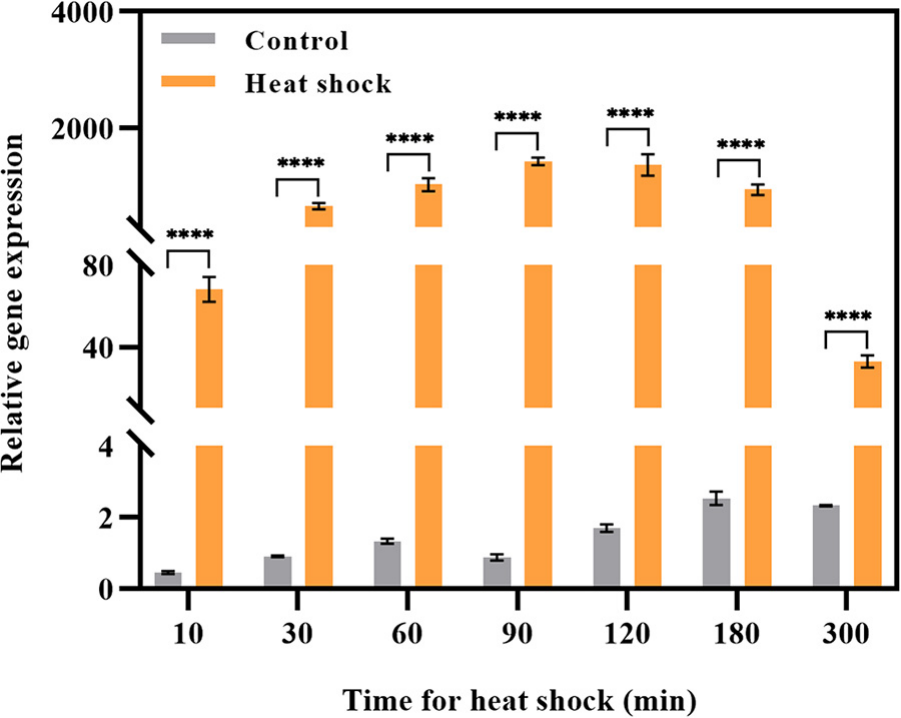
RT-qPCR analysis of the *hsp17* gene after heat shock at 45°C for 10 to 300 min. ****, *P < *0.0001.

The expression pattern of the *hsp17* gene was further confirmed by reverse transcription-quantitative PCR (RT-qPCR). When cells were exposed to 45°C, *hsp17* expression was approximately 147-fold higher than that in the control after only 10 min of heat shock (Fig. 3). As the duration of heat shock increased, *hsp17* expression overall initially increased and then decreased, culminating in a peak value at 90 min, which was almost 1,623-fold higher than that at 30°C. It remained relatively constant after 30, 60, and 120 min of heat shock and was approximately 772-fold higher than in the control. However, the expression level abruptly decreased and was only 13-fold higher than that at 30°C when the heat shock period was prolonged to 300 min. This result indicated that high temperature was an important factor inducing *hsp17* gene expression.

**FIG 4:**
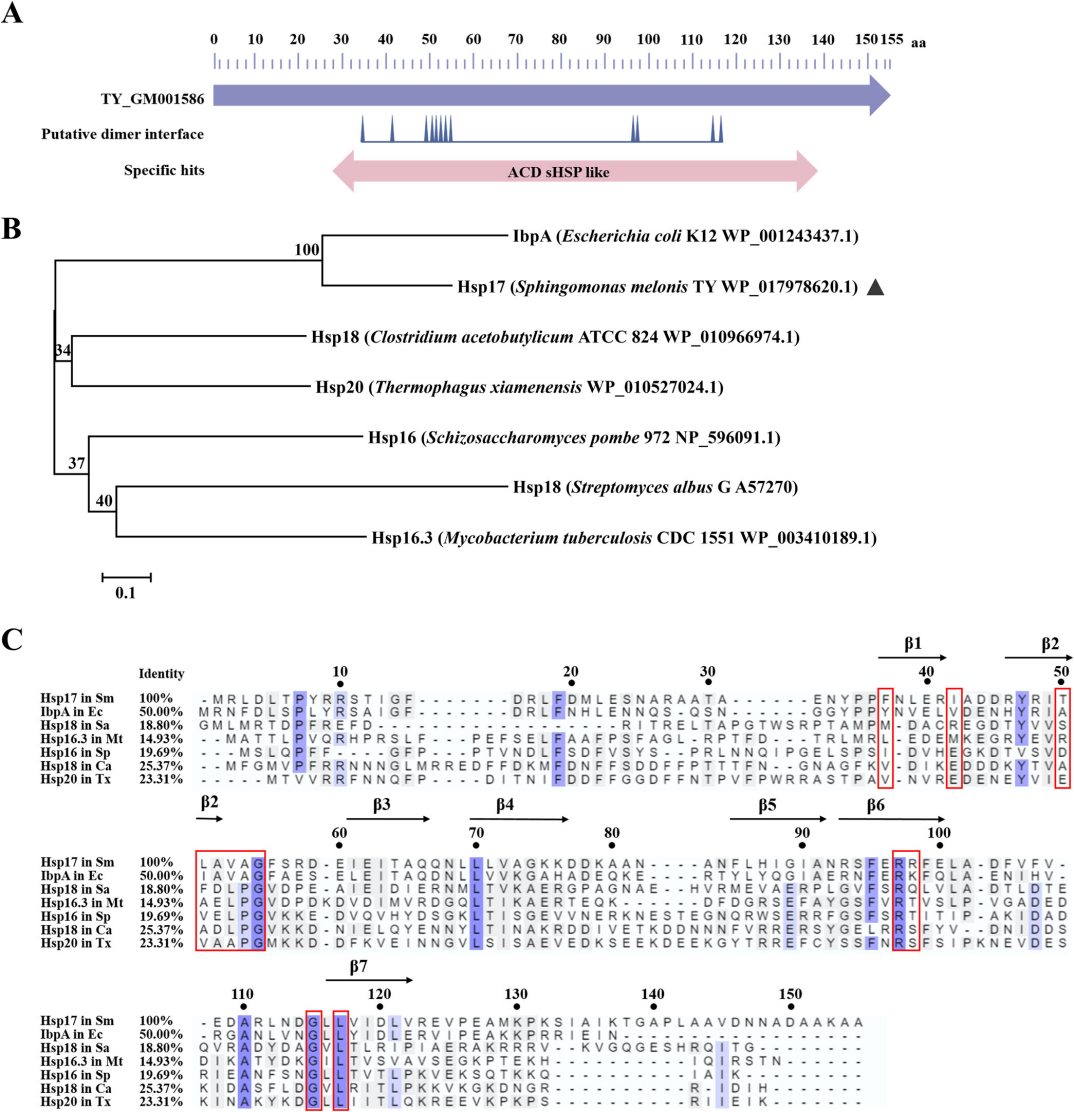
Sequence and structural analysis of the BJP26_07370 gene product of Sphingomonas melonis TY, Hsp17. (A) GenPept database analysis results of the BJP26_07370 gene product. (B) Phylogenetic tree of Hsp17. Hsp17 is marked with a black triangle. The numbers in the phylogenetic tree represent the reliability of each branch. (C) Amino acid sequence analysis of Hsp17 with other sHSPs. The shaded regions are the conserved feature regions in most sHSP family members. The sequence in the rectangle is the putative dimer interface. Sm, Sphingomonas melonis TY; Ec, Escherichia coli K-12; Sa, Streptomyces albus G; Mt, Mycobacterium tuberculosis CDC1551; Sp, Schizosaccharomyces pombe 972; Ca, Clostridium acetobutylicum ATCC 824; Tx, Thermophagus xiamenensis. The black arrows represent β-pleated sheets.

NCBI (http://www.ncbi.nlm.nih.gov/) GenPept database analysis of the Hsp17 primary amino acid sequence showed two conserved regions: an α-crystallin domain (ACD) of the α-crystallin type and a dimer interface region (Fig. 4A). The PROSITE database predicted the conserved structural domain to extend from 28 to 139 amino acids, since ACD is a conserved feature of the sHSP family. Amino acid sequence homology varied considerably. The degrees of homology between Hsp17 of *S. melonis* TY and IbpA of the type strain Escherichia coli K-12 (25), Hsp18 of Clostridium acetobutylicum ATCC 824 (26), Hsp16 of Schizosaccharomyces pombe 972 (27), Hsp18 of Streptomyces albus G (28), and Hsp16.3 of Mycobacterium tuberculosis CDC1551 (29) were 50.00%, 25.37%, 19.69%, 18.80%, and 14.93%, respectively (Fig. 4B and C). Compared with the Hsp20 family protein of the thermophile Thermophagus xiamenensis, Hsp17 showed 23.31% amino acid sequence homology. Another conserved region, the dimer interface area, was a polypeptide binding site composed of 12 residues (Fig. 4C, shown in a rectangle).

The conserved crystalline domain, consisting of a β-sandwich structure composed of 7 or 8 antiparallel β-sheets, is a shared structural characteristic of sHSPs (30). Homology models of Hsp17 were generated in SWISS-MODEL using sequence alignment, which clearly indicated the β-sandwich structure formed by seven antiparallel β-sheet structures (Fig. 4C; Fig. S5). The N- and C-terminal regions on the N and C sides of ACD played a role in the flexibility of the sHSP structures by being flexible in length and sequence (12). Similar to other sHSPs (27, 31), Hsp17 of *S. melonis* TY may exist as a polymer, such as a dimer, in cells.

### Hsp17 plays an essential role in cell viability.

As noted above, high temperature induced *hsp17* gene expression, and superhigh *hsp17* expression was observed throughout the heat shock period. New questions were thus raised, namely, whether *hsp17* knockout or overexpression impacted the ability of *S. melonis* TY to tolerate high temperatures. In addition, we also sought to address the question of whether the heterologous expression of *hsp17* could confer the ability to tolerate high temperatures in other organisms. Thus, the effect of *hsp17* on the cell viability of *S. melonis* TY and heterologous expression in E. coli DH5α was studied further based on analysis of CFU.

**FIG 5:**
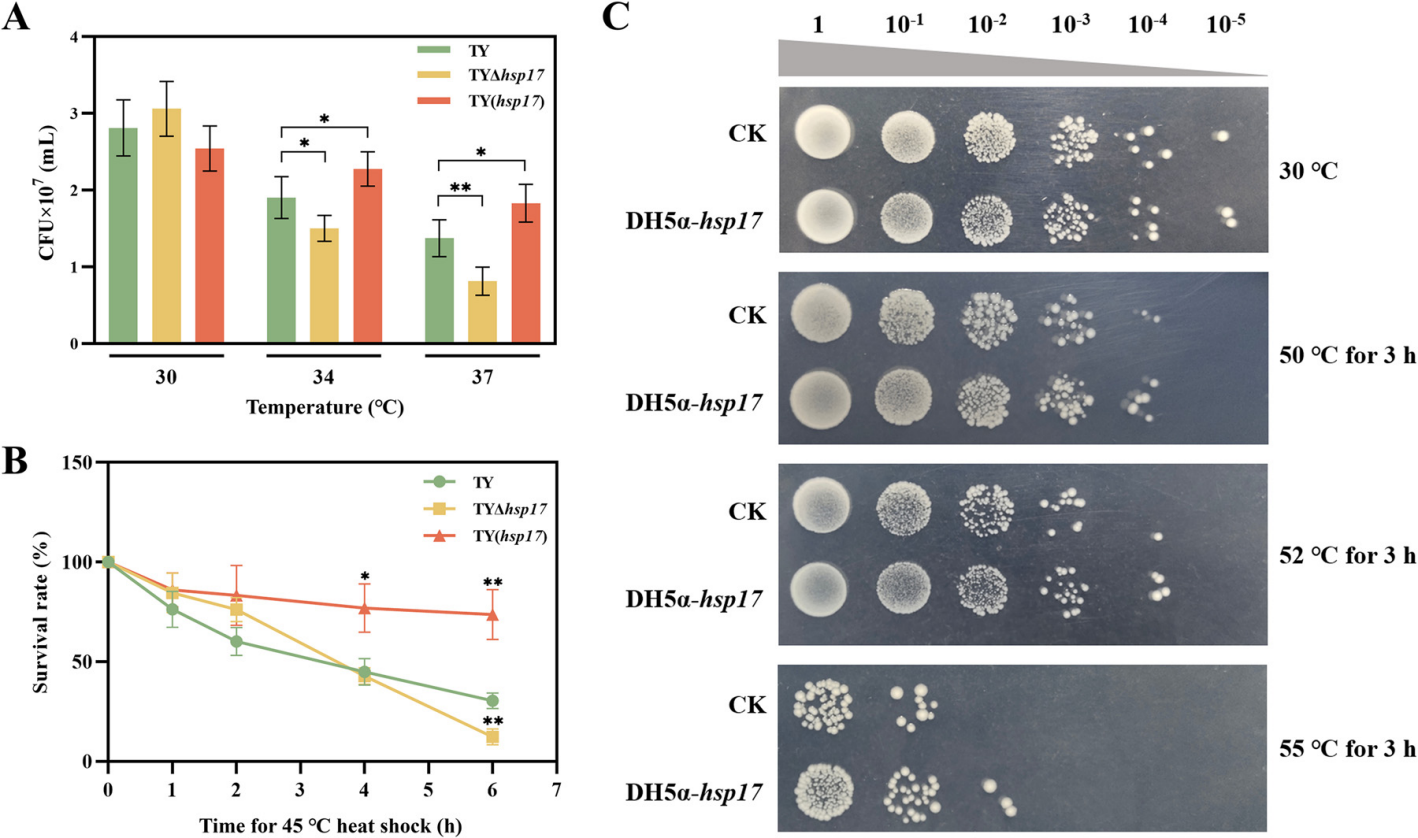
Function of *hsp17* in *S. melonis* TY and E. coli DH5 under heat stress conditions. (A) CFU counts of TY, TYΔ*hsp17*, and TY(*hsp17*) cells when incubated at different temperatures. *, *P < *0.05, **, *P < *0.01. (B) The survival rates of TY, TYΔ*hsp17*, and TY(*hsp17*) after 1, 2, 4, and 6 h of heat stress at 45°C. The data were collected with five replicate measurements per sample. *, *P < *0.05, **, *P < *0.01. (C) Effect of *hsp17* heterologous expression in E. coli DH5α on cell viability under heat stress conditions. CK, E. coli DH5α-pHGC02.

The function of *hsp17* in determining cell viability in *S. melonis* TY after heat shock was assessed with strains TY (wild type), TYΔ*hsp17*, and TY(*hsp17*). As shown in Fig. 5A, the numbers of CFU were essentially the same in these three strains when incubated at 30°C (approximately 2.81 × 10^7^ cells in 1 mL of resuspended sample). As expected, bacterial growth was inhibited as the temperature increased. We found that TYΔ*hsp17* lost the ability to withstand high temperatures, especially at 37°C. More TY(*hsp17*) cells than TY cells grew at 34°C and 37°C on solid medium, with approximately 1.20-fold and 1.33-fold more TY(*hsp17*) cells than of TY cells, respectively. Subsequently, we compared cell viabilities after exposing the cells to a temperature of 45°C for 1 to 6 h and found that TYΔ*hsp17* and TY(*hsp17*) showed higher heat sensitivity than the wild-type cells, especially after 4 to 6 h of heat stress (Fig. 5B). After 4 h of heat shock, the survival rate of TY(*hsp17*) was approximately 76.90%, while that of TY was only 42.77%. The difference was more obvious when the heat stress time was prolonged to 6 h. The survival rate of TY(*hsp17*) remained at 73.66% even after this extended period of heat stress, and this rate was approximately 2.42-fold that of TY, whose survival rate fell to 30.40%. Notably, the activity of TYΔ*hsp17* decreased with prolonged treatment time, and the difference became significant after 6 h of heat shock.

The capacity to resist heat stress was also imparted by the heterologous expression of *hsp17* in E. coli DH5α. When E. coli (*hsp17*) was cultured on agar medium at the optimum temperature, its growth was identical to that of wild-type E. coli at 37°C (Fig. 5C). Cell viability decreased in both E. coli and E. coli (*hsp17*) with increasing temperature. There were more surviving E. coli (*hsp17*) cells than wild-type cells after the temperature was increased to 55°C for 3 h of heat shock. The overexpression of *hsp17* enabled the E. coli DH5α strain to grow even at a 10^−2^ dilution at 55°C for 3 h, while no E. coli survived at the 10^−2^ dilution.

### Overexpression of *hsp17* maintains the normal morphology of *S. melonis* TY at high temperatures.

**FIG 6:**
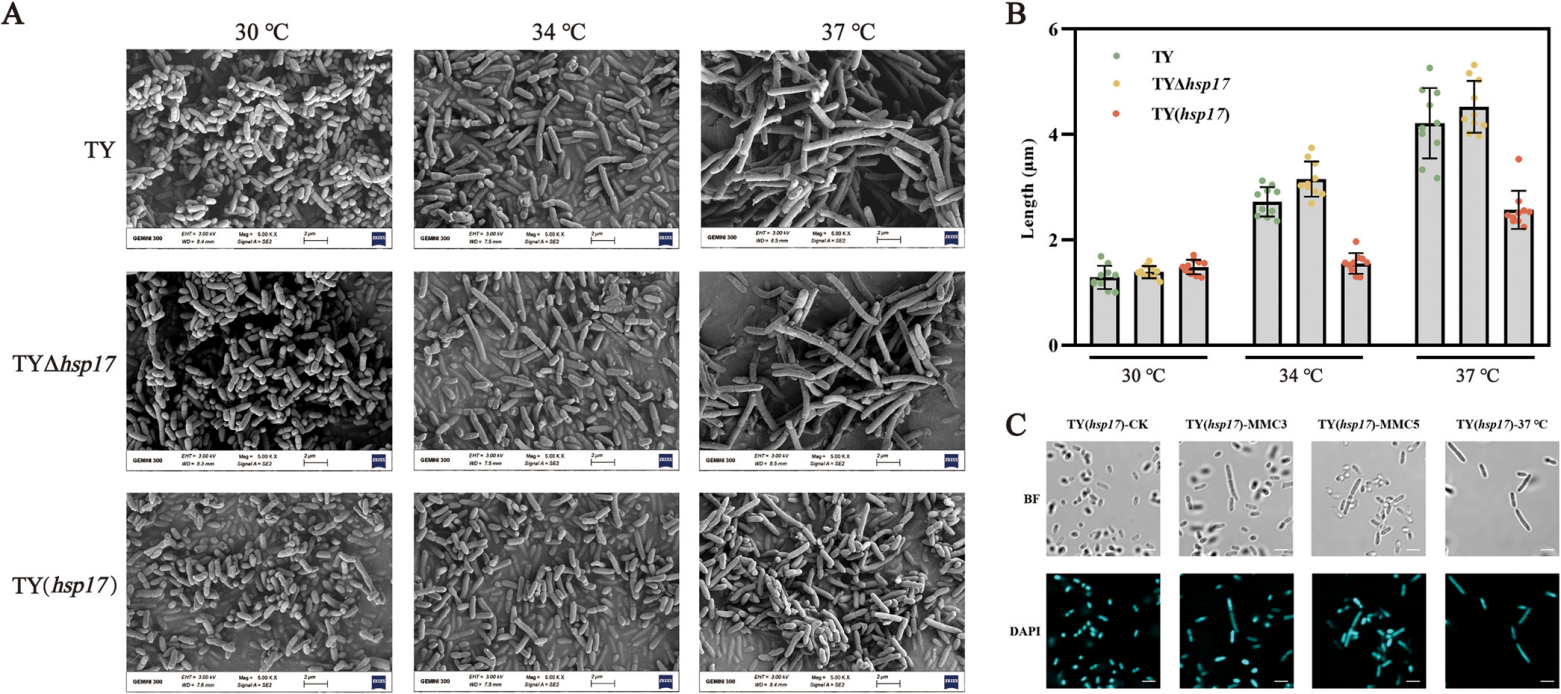
Images of morphology and statistical analysis of cell length. (A) SEM images of the TY, TYΔ*hsp17*, and TY(*hsp17*) strains cultured at 30°C, 34°C, and 37°C. (B) Statistical representation of the length of cells incubated at different temperatures. (C) Images of DAPI staining in TY(*hsp17*) cells treated with MMC or cultured at 37°C (MMC3, 3 μg · mL^−1^ MMC; MMC5, 5 μg · mL^−1^ MMC). The bar at the bottom right of each figure represents 2 μm (×600 magnification; BF, bright field; DAPI, DAPI staining).

Subsequently, scanning electron microscopy (SEM) was used to observe changes in cell morphology. The TY, TYΔ*hsp17*, and TY(*hsp17*) strains all showed similar morphologies at 30°C, with an average length of 1.39 μm and a short rod-like shape (Fig. 6A). The morphology of TY and TYΔ*hsp17* was elongated, and these strains formed filaments, with increased cell-cell adhesion as the temperature increased. At 34°C, TY and TYΔ*hsp17* cells were 1.12- and 1.27-fold longer, respectively, than those at 30°C, and when the temperature increased to 37°C, the cells were 2.28- and 2.26-fold longer than those at 30°C (Fig. 6B). To our surprise, TY(*hsp17*) had a length of approximately 1.48 μm at 30°C, which increased to only 1.55 μm and 2.57 μm at 34°C and 37°C, respectively, representing merely 0.05-fold and 0.74-fold increases over the length at 30°C (Fig. 6B). These results were confirmed through DAPI (4′,6-diamidino-2-phenylindole) staining of the cells (Fig. S6A). We also tested a further increase in the temperature, but the cells were unable to proliferate when the temperature was higher than 37°C.

Mitomycin C (MMC) can trigger the bacterial SOS response and directly affect cell division, as reported in E. coli and Bacillus subtilis (32, 33). Therefore, we examined whether the cells of *S. melonis* TY could be elongated during the SOS response by adding MMC. There was no significant effect of different concentrations of MMC on cell length. The morphology of TY and TYΔ*hsp17* was affected by MMC induction, and the cells took on a filamentous appearance (Fig. S6B and C). In contrast, TY(*hsp17*) was less sensitive to MMC, and different concentrations of MMC had little effect on the cell length. Notably, the morphology of TY(*hsp17*) cells grown at 37°C was comparable to that of TY(*hsp17*) cells treated with MMC (Fig. 6C).

## DISCUSSION

Microorganisms must adapt to their living environment to function normally, and the ability to overcome stressful conditions is critical. With the rapid development of sequencing technology, multi-omics analysis is now becoming increasingly common and efficient. Many heat shock proteins have been reported based on transcriptomic or proteomic analysis under heat stress (6, 14, 34). *Sphingomonas* species are widely existing microbes in the environment and play a vital role in pollutant degradation and plant protection; however, their response to heat shock and the ecological roles and functions of their HSPs have seldom been studied. In this study, we found that heat shock rapidly affected both gene transcription and translation in *S. melonis* TY, especially in terms of protein synthesis. A novel sHSP named Hsp17 was identified based on coexpression analysis of the transcriptome and proteome. As members of the HSP family, sHSPs usually function as “first aiders” in the cellular response to heat stress (12). We further investigated the sHSPs of *Sphingomonas* and explored their potential physiological functions, which may provide new insight into the *Sphingomonas* heat stress response. We believe that Hsp17 acts as a chaperone and plays a positive role in the response to heat stress. To test this hypothesis, the TYΔ*hsp17* strain and an *hsp17* overexpression strain were constructed. We found that deletion of *hsp17* affected the heat resistance of *S. melonis* TY, while overexpression of *hsp17* facilitated cell growth and maintained cell morphology under high temperatures (Fig. 5 and 6).

The heat stress response is triggered by an increase in the ambient temperature of only a few degrees, as reported in some thermophilic bacteria (35). Proteins are optimized during evolution to remain only marginally stable at their respective optimal growth temperatures. Even a small increase in temperature can lead to protein misfolding, entanglement, and nonspecific aggregation (36). The ribosome is an essential intracellular organelle not only for protein synthesis but also as the cellular thermosensor of prokaryotic bacteria (37). In the present study, several genes encoding ribosomal proteins, including the 50S ribosomal proteins L2 (*rplB*), L12 (*rplL*), and L29 (*rpmC*) and the 30S ribosomal proteins S3 (*rpsC*) and S17 (*rpsQ*), were significantly downregulated under heat shock conditions at the mRNA level in *S. melonis* TY (see Fig. S3 in the supplemental material). This downregulation could result in the destabilization of ribosomal subunits when cells are exposed to high temperatures. The number of ribosomal units determines the rate of protein synthesis associated with cellular growth, and the synthesis of an intact ribosome is an energy-demanding process (38, 39). We speculate that *S. melonis* TY responds to heat shock by reducing its rate of protein synthesis required for cellular growth to reduce energy metabolism. In addition, we found that for the vast majority of transcripts showing increased levels in response to heat shock, no elevation of corresponding protein levels was observed (Fig. 2B and C), similar to previously reported results (6, 40, 41). It is conceivable that protein production can be offset by stress-induced protein degradation, and the rate of production equals the rate of degradation and therefore shows no net change (6). Thus, heat stress is often associated with a high level of transcriptional regulation in comparison to nonstress situations.

As ubiquitous molecular chaperones, sHSPs generally function as large oligomers consisting of multiple subunits that can suppress protein aggregation and protect against cell stress. In this study, a novel sHSP of 17.2 kDa with the conserved α-crystallin domain of sHSP families, designated Hsp17, was identified through multi-omics analysis, and it was presumed to be active as a dimer. The *hsp17* gene was found to be sensitive to heat stress, and its transcriptional level increased after only 10 min of exposure to heat stress (Fig. 3). The effect of Hsp17 on cell viability and stress resistance against heat was confirmed in our study, and the main findings were consistent with those of previous studies (14, 27). Since Hsp17 shared 44.9% identity with IbpA at the amino acid level (Table 2), we speculated that Hsp17 may have biological functions similar to those of IbpA in cellular stress defense. The heat resistance of *S. melonis* TY, including both its thermotolerance and thermosensitivity, was severely compromised by the deletion of *hsp17* (Fig. 3A and B). In E. coli, IbpA and IbpB mutants exhibit growth defects at 46°C (42). Besides, an IbpA deletion mutant of Pseudomonas putida also showed a severe growth defect under heat stress conditions (43). Thus, the absence of sHSPs may constitute loss of the first line of cellular defense against heat stress, leading to disorders of physiological function, abnormal metabolism, and even cell death. Compared with TY or TYΔ*hsp17* cells, the overexpression of *hsp17* conferred heat tolerance (Fig. 3A and B). Thus, we believe that sHSPs play an important role in bacterial stress tolerance. These advantages may be particularly beneficial for promoting host symbiotic colonization and contaminated site niche competition in natural settings.

Bacteria display a variety of cell shapes, including round, rod, spiral, and amorphous morphologies (44). However, under stress induced by environmental pressure, adaptive bacteria undergo physiological and morphological changes (45–47). In our study, when cells were incubated at 30°C, they maintained their rod shape, with a length of 1.39 μm (Fig. 6A and B). As the temperature increased, the morphological transition in *S. melonis* TY became pronounced: cells elongated and remained connected, forming multicellular chains or filaments (Fig. 6A), as confirmed in other studies (48, 49). The deletion of the *hsp17* gene did not affect cell morphology compared with that of wild-type cells, possibly due to compensatory mechanisms. However, overexpression of the *hsp17* gene greatly decreased the tendency of cells to elongate (Fig. 6A and B). This finding could be explained by the proposed bacterial suicidal response hypothesis for the destruction and cessation of cell division under high-temperature conditions (50, 51). To verify that the filamentous morphology of the cells was indeed caused by abnormal division, MMC, which can prevent cell division and act as a DNA cross-linker, was selected for cell treatment (52, 53). MMC clearly induced cell elongation in both TY and TYΔ*hsp17* cells, but this effect was attenuated by the overexpression of *hsp17* (Fig. 6C). Therefore, we speculated that high temperature affected the expression of genes involved in cell division and caused an abnormal cell shape in *S. melonis* TY. This morphological change may be an adaptive mechanism to combat and survive under stressful environmental conditions. However, the overexpression of *hsp17* mitigated DNA damage and maintained the correct folding of related proteins under high temperature, which is important for maintaining cellular homeostasis.

Extremely heterogeneous sHSPs constitute the most widespread family of molecular chaperones, present in all kingdoms of life (11). In the present study, the importance of a novel sHSP, Hsp17, in stress resistance and in maintaining cell shape under high temperature was demonstrated. Moreover, the heterologous expression of *hsp17* conferred more resistance to heat stress in E. coli. According to the concept of synthetic biology, a large fraction of the nucleic acid sequences in the “biological part” are genes that encode proteins (54, 55). Compared with other induction systems, temperature control components have the advantages of low cost, fast responses, and removability (56). We believe that with further study of sHSPs, the elucidation of additional mechanisms will enhance our understanding of how microbes adapt themselves to tolerate such extreme environments. Furthermore, we think that the functions of *hsp17* are consistent with the core principles of synthetic biology. Similarly, when *hsp17* is present or even overexpressed, it can play an important role in microbial assembly as a heat resistance element, reducing the metabolic burden of cells and providing a reference for enabling heat resistance in chassis microbial cells.

## MATERIALS AND METHODS

### Bacterial strains, plasmids, and growth conditions.

The bacterial strains and plasmids used in this study are listed in Table 3, and the primers are shown in Table 4. The wild-type strain *S. melonis* TY and its derivatives were cultured aerobically in modified LB medium without NaCl (10 g · L^−1^ tryptone, 5 g · L^−1^ yeast extract) at 30°C. E. coli DH5α and derivatives were routinely grown at 37°C in LB medium (10 g · L^−1^ tryptone, 5 g · L^−1^ yeast extract, 10 g · L^−1^ NaCl). Cell densities were monitored by measuring the optical density at 600 nm (OD_600_) using a Lambda25 spectrophotometer. When necessary, kanamycin and tetracycline were used at final concentrations of 50 and 10 μg · mL^−1^, respectively. DAPI was used at a final concentration of 0.3 mM in E. coli WM3064. Competent E. coli WM3064 cells were prepared according to standard methods using 0.1 M CaCl_2_ in 20% (vol/vol) glycerol (57).

**TABLE 3:** Strains and plasmids used in this study

Strain or plasmid	Characteristics^*a*^	Reference or source
Strains		
Escherichia coli		
DH5α-pHGC02	DH5α transformed with pHGC02, Tet^r^	This study
DH5α-*hsp17*	DH5α transformed with pHGC02-*hsp17*, Tet^r^	This study
WM3064	Donor strain for conjugation, DAPI auxotroph	Laboratory stock
*Sphingomonas* species		
TY	Wild type transformed with pHGC02, G–, Tet^r^, Amp^r^, Kan^s^	This study
TYΔ*hsp17*	TY mutant with *hsp17* gene replaced by Kan resistance gene from plasmid pEX18Tc, Tet^r^, Kan^r^	This study
TY(*hsp17*)	TY mutant with *hsp17* gene overexpression with plasmid pHGC02, Tet^r^	This study
Plasmids		
pEX18Tc	Gene knockout vector, Tet^r^	Laboratory stock
pEX18Tc-*hsp17*	*hsp17* gene knockout vector containing two DNA fragments homologous to the upstream and downstream regions of the *hsp17* and kanamycin resistance gene	This study
pHGC02	Overexpression vector, Tet^r^	Laboratory stock
pHGC02-*hsp17*	Overexpression vector for *hsp17* by cloning *hsp17* into the EcoRI-HindIII restriction site, Tet^r^	This study

a Tet^r^, tetracycline resistance; Amp^r^, ampicillin resistance; Kan^s^, kanamycin sensitivity; Tet^s^, tetracycline sensitivity; G–, Gram-negative bacterium.

**TABLE 4:** Primers used in this study

Primer	Sequence (5′–3′)	Purpose
Kan-F	TGTCTCAAAATCTCTGATGTTAC	To amplify kanamycin resistance gene from pTnMod-Okm for gene knockout
Kan-R	TTAGAAAAACTCATCGAGCATC	
*hsp17*-up-F	ACATGATTACGAATTCATTGCCGGCGGTCAGCTCGA	To amplify upstream fragment of *hsp17* for gene knockout
*hsp17*-up-R	AGAGATTTTGAGACAGTCAAATCCTCCGAACGAAG	
*hsp17*-down-F	GATGAGTTTTTCTAAGTCGACGTAGCACTTCCTTA	To amplify downstream fragment of *hsp17* for gene knockout
*hsp17*-down-R	GGCCAGTGCCAAGCTTGCGATGAAGGGGGCAAAACC	
*hsp17*-VF1	GTAATTGCCGGCGGTCAGCT	To verify strain TYΔ*hsp17* by PCR or sequencing
*hsp17*-VR1	GAAACAACTCTGGCGCATCG	
*hsp17*-VF2	GCTTAACGCGATGTTGGCGG	
*hsp17*-VR2	CGATGCGCCAGAGTTGTTTC	
pEX18Tc-VF	AGCTGGCACGACAGGTTTCC	To verify construction of pEX18Tc-related vectors by PCR or sequencing
pEX18Tc-VR	GCGCTACGGCGTTTCACTTC	
pHGC02-h*sp17*-F	TCACACAGGAGAATTCATGCGACTCGACCTGACCCC	To amplify the fragment of *hsp17* for gene overexpression
pHGC02-h*sp17*-R	CAAAACAGCCAAGCTTTTACGCAGCCTTGGCGGCGT	
pHGC02-F	GGCGGCACCTCGCTAACGGAT	To verify the overexpression of *hsp17* by PCR or sequencing
pHGC02-R	GCCAGGCGGTGAAGGGCAAT	
*hsp17*-SYBR-F	GGCAAGAAGGACGACAAG	To amplify 96-bp fragment of *hsp17* for qPCR
*hsp17*-SYBR-R	CACGAAATCGGCGAGTTC	
338F	CCTACGGGAGGCAGCAGCAG	To amplify V3 region in 16S RNA gene for qPCR
518R	ATTACCGCGGCTGCTGG	

### RNA extraction.

Total bacterial samples were collected, mixed with 0.2 volumes of stop-mix (95% ethanol and 5% phenol, vol/vol), and snap-frozen in liquid nitrogen. Pellets were resuspended in 100 μL lysozyme solution (15 mg · mL^−1^ lysozyme in Tris-EDTA [TE] buffer, pH 8.0). Total RNA was isolated with the Omega E.Z.N.A. bacterial RNA kit.

### RNA-seq analysis of *S. melonis* TY in response to heat shock.

For the transcriptomic sequencing (RNA-seq) analysis, *S. melonis* TY was cultured in modified LB medium at 30°C for 10 h. Then, one sample was transferred to and maintained at 45°C for heat shock analysis, and the other sample (30°C) was used for non-heat shock analysis. The cells were treated for 10 min or 30 min and then immediately harvested by centrifugation at 12,000 × *g* for 5 min at 4°C. The supernatant was discarded, and the cell pellets were frozen in liquid nitrogen for 10 min. The transcriptomic analysis of the samples was performed by Beijing Novogene Bioinformatics Technology Co., Ltd. (China). A total of 15 samples (5 treatments with 3 replicates) were sequenced. After removing the sequencing adapters and trimming consecutive low-quality bases (quality, <20) from the reads, high-quality RNA-seq reads were aligned, and gene expression abundance was quantified by the fragments per kilobase of exon per million fragments mapped (FPKM) method (58). Genes with a *P* value of <0.05 and log_2_ |(fold change [FC])| of >0 were classified as differentially expressed. RNA-seq analysis was performed essentially as described previously (20).

### Proteomic analysis.

Cells were cultured in modified LB medium at 30°C for 10 h. One sample (10 mL) was transferred to and maintained at 45°C for 30 min for heat shock analysis, and the other sample (10 mL, 30°C) was used for non-heat shock analysis. Each treatment included three biological repeats. The tandem mass tag (TMT) label quantitative proteomics project was completed in cooperation with Shanghai Applied Protein Technology Co., Ltd. (China). Samples were then subjected to the following steps: protein extraction and digestion, TMT labeling of peptides, high-pH reversed-phase fractionation, liquid chromatography-mass spectrometry analysis, and identification and quantitation of proteins. Only proteins with a FC of >1.2-fold (more than 1.2-fold upregulation or less than 0.83-fold downregulation) and a *P* value of <0.05 (*t* test or other) were considered to show statistically significant differences. The proteomic analysis was performed essentially as described previously (20).

### RT-qPCR validation.

RT-qPCR assays were performed using total RNA preparations obtained from three independent cultures. The RNA was reverse transcribed into cDNA using the PrimeScript RT reagent kit (TaKaRa). The primers were designed with Beacon Designer software based on the full genome sequence of *S. melonis* TY (Table 4). The PCR assays were carried out with TransStart top green qPCR super mix using a three-step method on a Qiagen Rotor-Gene Q detection system (Germany) according to the manufacturer’s recommendations. The 16S V3 rRNA gene was used as the endogenous reference control, and relative gene expression levels were determined using the comparative threshold cycle (average amplification efficiency)-CT method (59). The data were analyzed using LinRegPCR software (60).

### Gene knockout and overexpression.

The in-frame disruption of *hsp17* in *S. melonis* TY was performed using the suicide plasmid pEX18Tc and a two-step homologous recombination method (61). The pEX18Tc-*hsp17* plasmid for gene knockout was constructed by fusing the PCR products of the kanamycin resistance gene and two upstream and downstream fragments of the target gene, amplified with the primers shown in Table 4. The pHGC02-*hsp17* plasmid for gene overexpression was constructed by fusing the PCR products of the *hsp17* gene. After sequencing, the correct target monoclonal species was preserved.

### Heterologous expression of *hsp17*.

Fragments of *hsp17* were amplified using primers specific to the *S. melonis* TY genome (Table 4). The target band was purified and cloned into the pHGC02 vector (digested with EcoRI and HindIII) and transformed into E. coli DH5α cells. Then, single clones were screened on LB agar plates with tetracycline. Correct clones were confirmed by colony PCR and sequencing.

### Thermotolerance and thermosensitivity evaluation.

TY, TYΔ*hsp17*, and TY(*hsp17*) cells were incubated with shaking until the culture reached an OD_600_ of ~3 (~10 h), harvested by centrifugation at 7,000 × *g* for 5 min, and resuspended in phosphate-buffered saline (PBS) to an OD_600_ of ~0.05 in 1.5-mL Eppendorf (EP) tubes. Next, the cells were serially diluted in PBS (1 mL) from 1 to 10^−5^, and 100 μL of the cells at each gradient was spread evenly on a modified LB agar plate and incubated at 30, 33, or 37°C for 2 days. The plates were finally selected at the 10^−4^ dilution concentration for counting to evaluate thermotolerance. For the thermosensitivity test, TY, TYΔ*hsp17*, and TY(*hsp17*) cells were grown as described above. After the cell concentration was adjusted to an OD_600_ of ~0.05 (in 1.5-mL EP tubes), the cells were incubated in a 45°C metal bath incubator for 1, 2, 4, or 6 h without shaking. Each sample was serially diluted (10 times), spread onto an LB agar plate, and incubated at 30°C for 4 days. The survival rate was determined as follows: [(number of viable bacteria after treatment)/(number of untreated viable bacteria)] × 100%. Five biological replicates were established for each experiment. The E. coli DH5α cells were harvested by centrifugation at 7,000 × *g* for 5 min and resuspended in PBS to an OD_600_ of ~0.05. Subsequently, the cells were subjected to temperatures of 50, 52, and 55°C for 3 h. The bacterial cells (100 μL) were evenly spread on LB agar plates in serial 10-fold dilutions (1 to 10^−5^) and incubated at 37°C for 2 days.

### SEM.

Bacteria were cultured in LB at 30, 34, and 37°C, collected (OD_600_, ~0.6) by centrifugation, and washed 3 times with 0.1 M PBS buffer (pH 7.4). Following 2 h of fixation with 2.5% glutaraldehyde (diluted in 0.1 M PBS buffer), the samples were rinsed 3 times with the same buffer. After being postfixed with osmic acid, the samples were washed 3 times in 0.1 M PBS buffer. Gradient dehydration was performed in an ethanol solution series (30%, 50%, 70%, 80%, 90%, 95%, and 100%; 10 min in each step) and then critical point dried with liquid CO_2_. The samples were finally mounted on aluminum stubs, sputter coated with gold/palladium, and imaged using a G300 field emission SEM (Carl Zeiss, Germany).

### Microscopy monitoring of cells and nucleoids.

Cells were inoculated with an OD_600_ of ~0.05. After 8 h of shaking culture, MMC was used as an inducer at a concentration of 3 μg · mL^−1^ (MMC3) or 5 μg · mL^−1^ (MMC5) for 4 h of induction. The cells were harvested by centrifugation at 12,000 × *g* for 2 min. After removal of the supernatant, samples were fixed with ice-cold methanol for 10 min, washed 3 times with 0.1 M PBS, stained with DAPI for 5 min to fix cells on glass slides, and then observed by laser scanning confocal microscopy (FV-3000; Olympus, Japan).

### Data availability.

The transcriptome data have been deposited in the NCBI BioProject database under accession number PRJNA931796.

The MS proteomics data have been deposited in the ProteomeXchange Consortium via the iProX partner repository with the data set identifier PXD039912.
