# Supplementary figures and images for: Characterization of Hsp17, a Novel Small Heat Shock Protein, in Sphingomonas melonis TY under Heat Stress
Source: Microbiol Spectr. 2023 Jul 12;11(4):e01360-23. doi: 10.1128/spectrum.01360-23 (PMC10434288; doi:10.1128/spectrum.01360-23)

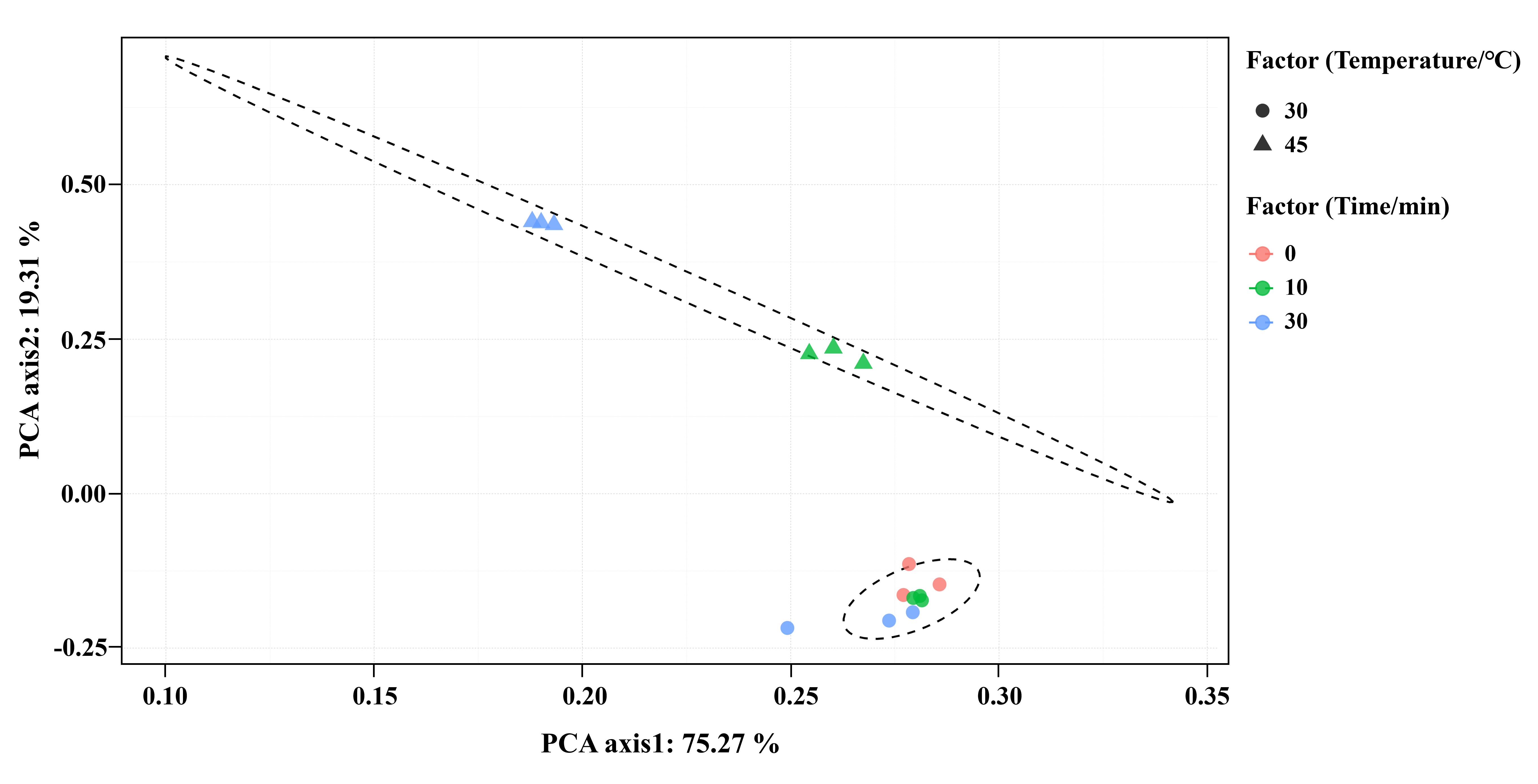

Supplement: Supplemental file 1 — Figure S1. Download spectrum.01360-23-s0002.tif, TIF file, 1.2 MB [file spectrum.01360-23-s0002.tif]

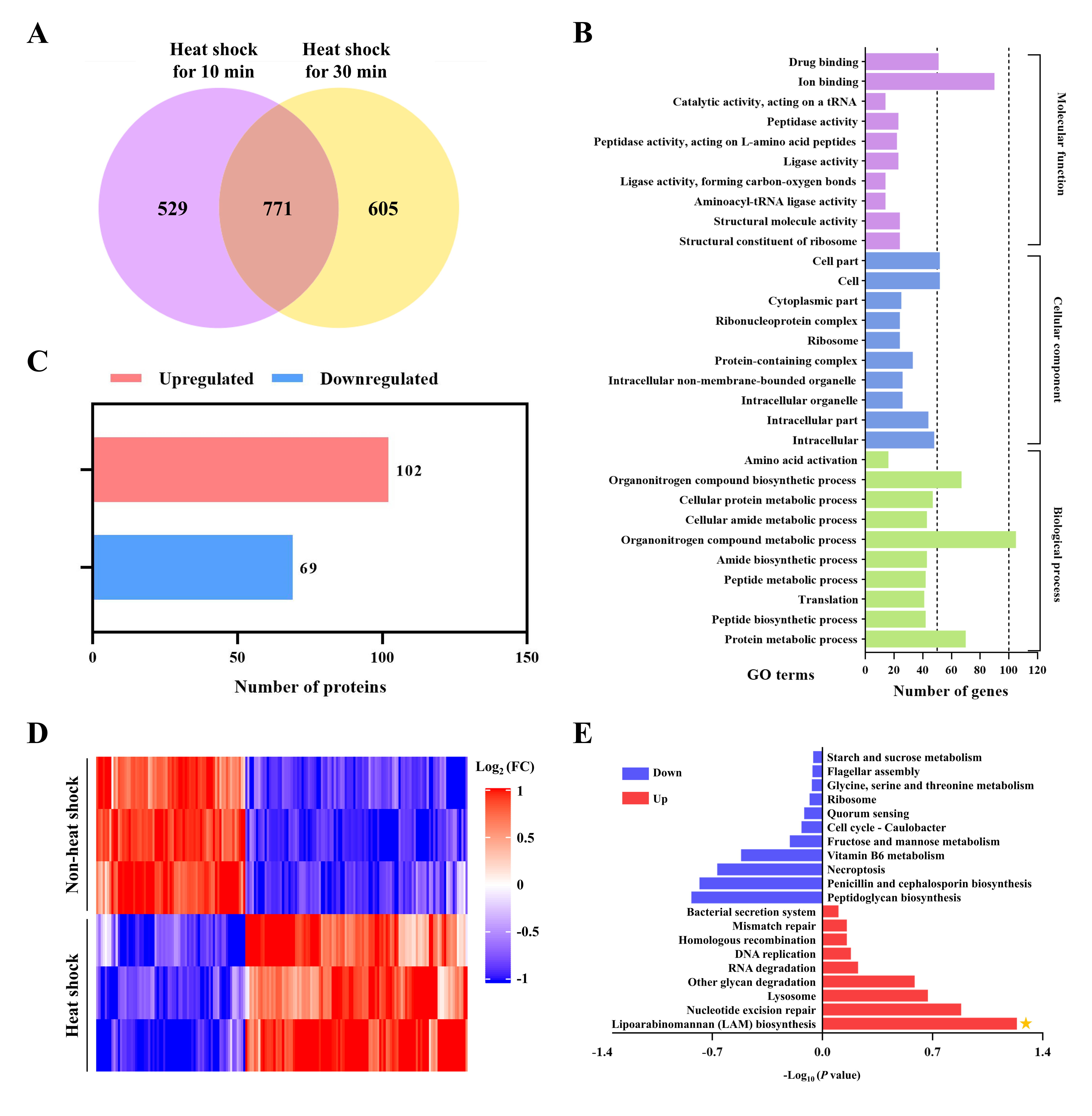

Supplement: Supplemental file 2 — Figure S2. Download spectrum.01360-23-s0003.tif, TIF file, 7.5 MB [file spectrum.01360-23-s0003.tif]

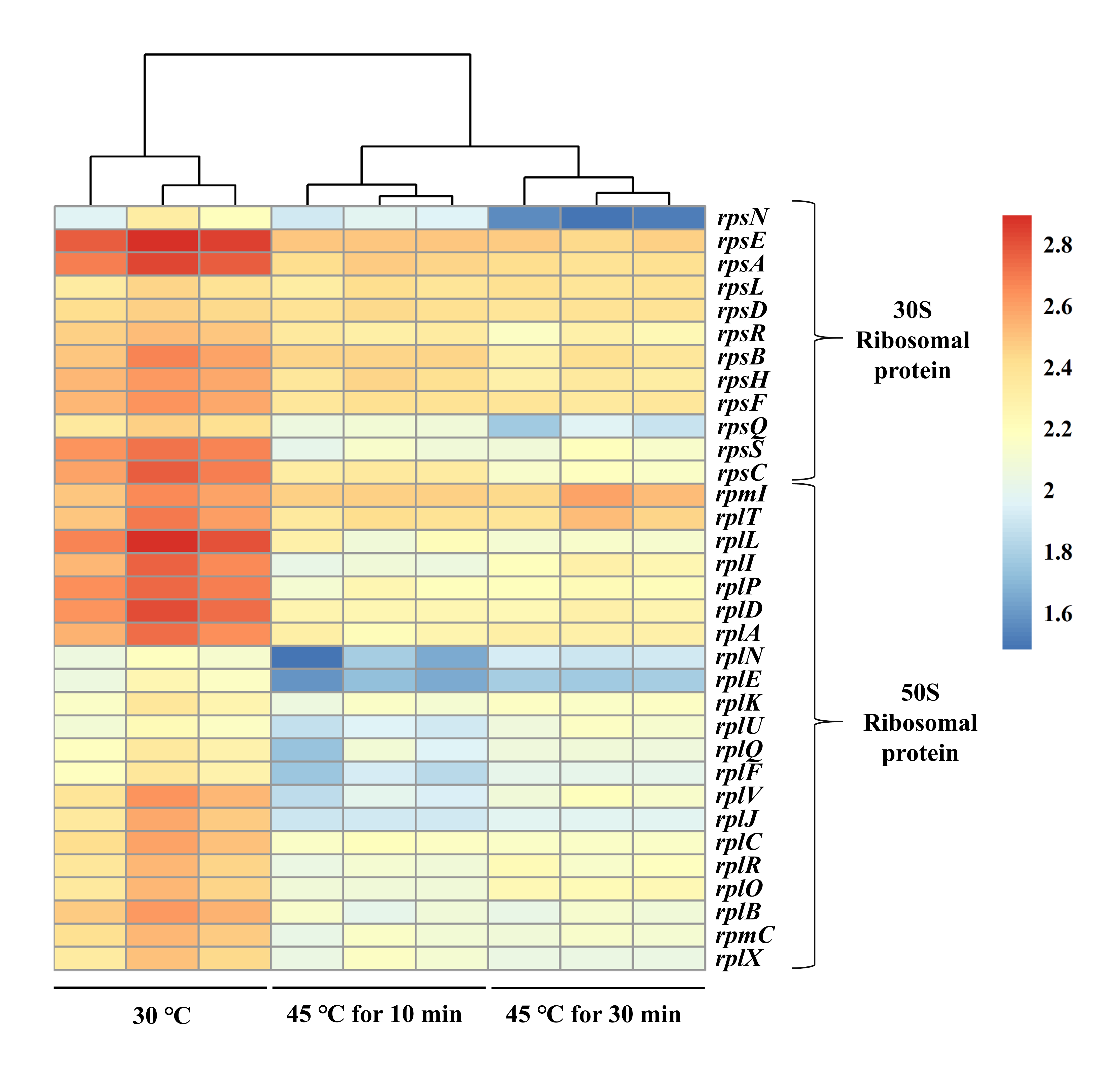

Supplement: Supplemental file 3 — Figure S3. Download spectrum.01360-23-s0004.tif, TIF file, 2.9 MB [file spectrum.01360-23-s0004.tif]

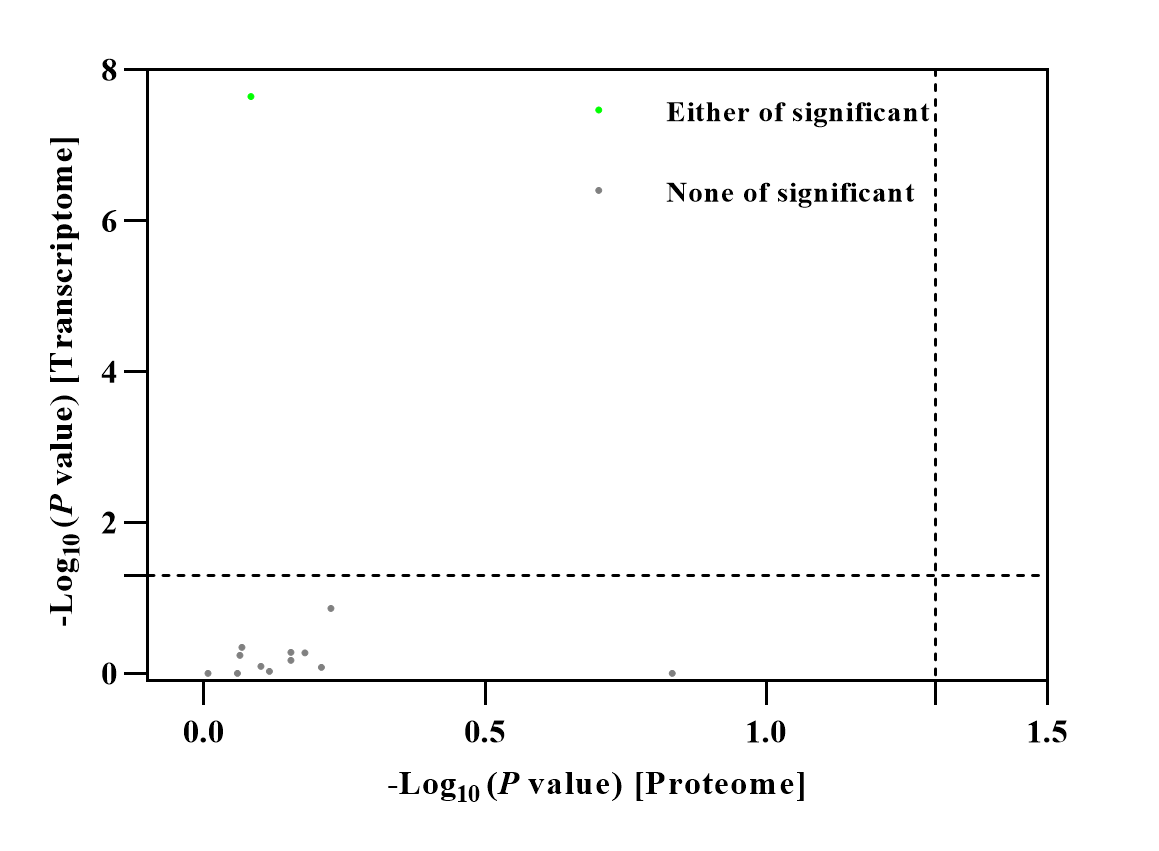

Supplement: Supplemental file 4 — Figure S4. Download spectrum.01360-23-s0005.tif, TIF file, 0.09 MB [file spectrum.01360-23-s0005.tif]

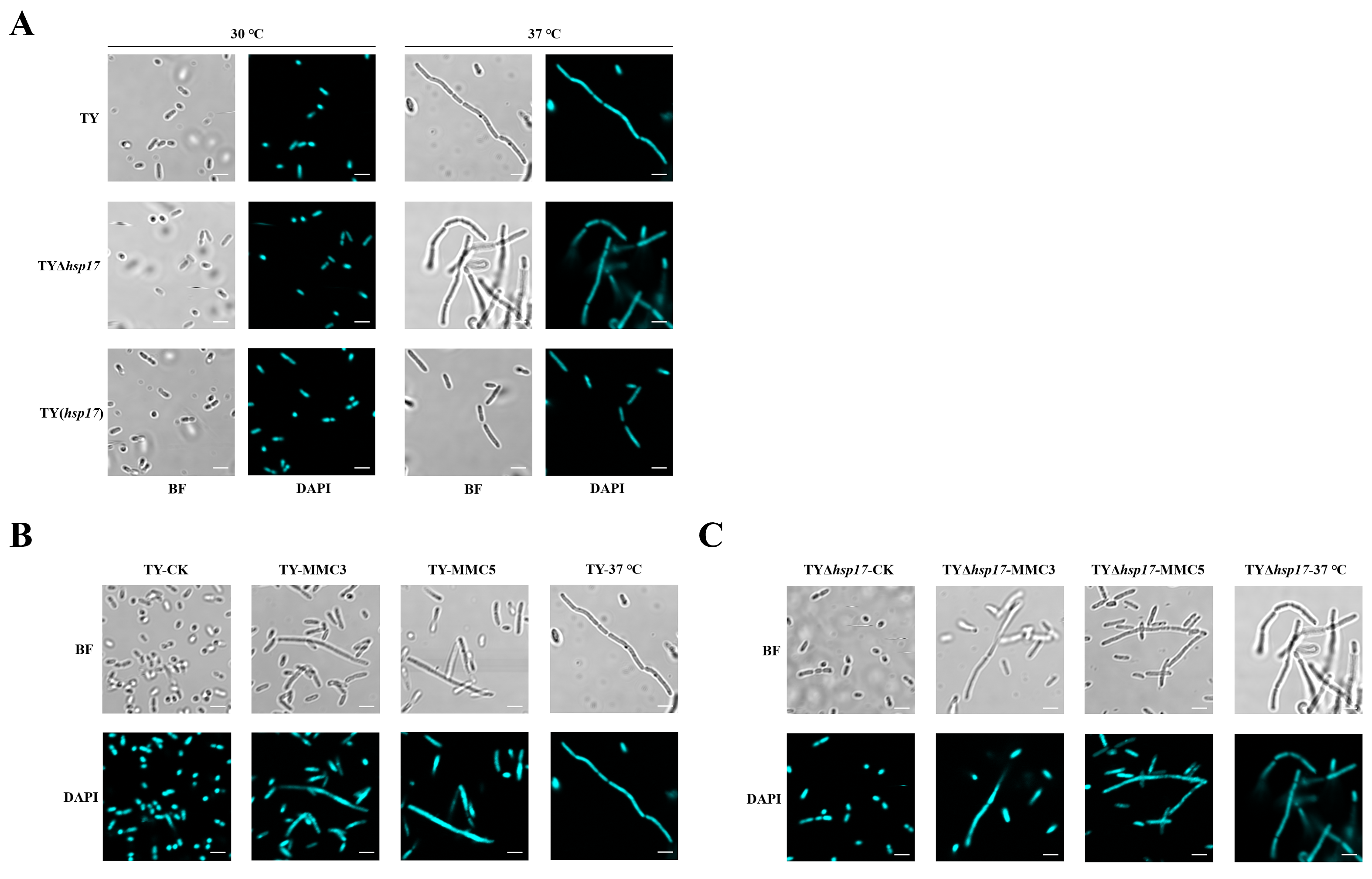

Supplement: Supplemental file 6 — Figure S6. Download spectrum.01360-23-s0001.tif, TIF file, 2.5 MB [file spectrum.01360-23-s0001.tif]
