# Supplementary material for: Characterization of Hsp17, a Novel Small Heat Shock Protein, in Sphingomonas melonis TY under Heat Stress
Source: Microbiol Spectr. 2023 Jul 12;11(4):e01360-23. doi: 10.1128/spectrum.01360-23 (PMC10434288; doi:10.1128/spectrum.01360-23)
Supplement: Supplemental file 7 — Supplemental material. Download spectrum.01360-23-s0007.docx, DOCX file, 0.03 MB [file spectrum.01360-23-s0007.docx]

**Supplementary data**

**The characterization of a novel small heat shock protein Hsp17 in *Sphingomonas melonis* TY under heat stress**

**Authors:**

Yihan Wang^a, b^, Xiaoyu Wang^a, b^, Hao Wu^a, b^, Lvjing Wang^a, b^, Haixia Wang^a^, Zhenmei Lu^a, b #^

^a^ MOE Laboratory of Biosystem Homeostasis and Protection, College of Life Sciences, Zhejiang University, Hangzhou 310058, China

^b^ Cancer Center, Zhejiang University, Hangzhou 310058, China

^#^Address correspondence to Zhenmei Lu, lzhenmei@zju.edu.cn

**Keywords**: Heat shock; Small heat shock protein; Coexpression analysis; Cell morphology; Stress resistance

Figure S1 Scatter plot of the two main factors identified from principal component analysis (PCA). RNAseq data of three biological replicates of each temperature and time combination. The X-axis shows principal component 1 (PCA axis1), and the Y-axis shows principal component 2 (PCA axis2). Dots represent individual biological replicates from the groups incubated at 30 ℃ for different times. Triangles represent individual biological replicates from the groups incubated at 45 ℃ heat shock for different times. Pink represents treatment for 0 min; green, treatment for 10 min; and blue, treatment for 30 min.

Figure S2 Heat shock induces changes in gene and protein expression. (A) Venn diagram analysis of DEGs after 10 min or 30 min of heat shock. Purple and yellow circles indicate the numbers of DEGs identified after 10 min or 30 min of heat shock, respectively. Overlapping areas represent genes that were differentially expressed at both time points. (B) GO enrichment of DEGs at both 10 min and 30 min after heat shock. (C) Statistics of DEPs under heat shock. (D) Heatmap of DEPs based on proteomic data. Samples treated at 30 °C or 45 °C for 30 min were used for analysis. (E) KEGG pathway enrichment analysis of DEPs. Asterisks indicate significant differences (*P* < 0.05).

Figure S3 Heatmap of DEGs enriched in the ribosome pathway after heat shock based on the KEGG database. The color scale represents FPKM normalized log10 transformed counts.

Figure S4 Enrichment correlation analysis of KEGG pathway. The green points represent pathways that were significantly enriched in either the proteome or the transcriptome, and the gray points represent pathways that were not significantly enriched in either the proteome or the transcriptome.

Figure S5 Predicted three-dimensional structure of protein Hsp17 by SWISS-MODEL.

Figure S6 DAPI staining under different treatment conditions. (A) DAPI staining of TY, TYΔ*hsp17* and TY(*hsp17*) cultured at 30 ℃ (left panel) or 37 ℃ (right panel). (B, C) DAPI staining of TY and TYΔ*hsp17* treated with different concentrations of MMC (MMC3: 3 μg·mL^-1^ MMC; MMC5: 5 μg·mL^-1^ MMC). The bar at the bottom right of the figure represents 2 μm (600 times magnification; BF, bright field; DAPI, DAPI staining).

Table S1 Coexpression analysis of transcriptome and proteome combined GO enrichment.

| GO_ID | Term | Category | Proteome *p* value | Transcriptome *p* value | Number of correlations |
| --- | --- | --- | --- | --- | --- |
| GO:0019725 | cellular homeostasis | P | 0.002212 | 0.003162022 | 2 |
| GO:0042592 | homeostatic process | P | 0.004805 | 0.00939234 | 2 |
| GO:0004519 | endonuclease activity | F | 0.009047 | 0.529441115 | 1 |
| GO:0065008 | regulation of biological quality | P | 0.062622 | 0.018307426 | 2 |
| GO:0004518 | nuclease activity | F | 0.10205 | 0.389409881 | 1 |
| GO:0017171 | serine hydrolase activity | F | 0.119806 | 0.015584216 | 1 |
| GO:0008236 | serine-type peptidase activity | F | 0.119806 | 0.015584216 | 1 |
| GO:0009605 | response to external stimulus | P | 0.154655 | 0.545306416 | 1 |
| GO:0009055 | electron transfer activity | F | 0.16602 | 0.00394537 | 1 |
| GO:0006508 | proteolysis | P | 0.230688 | 0.000788348 | 2 |
| GO:0070011 | peptidase activity, acting on L-amino acid peptides | F | 0.292924 | 0.002178671 | 2 |
| GO:0042221 | response to chemical | P | 0.332624 | 0.477684544 | 1 |
| GO:0043412 | macromolecule modification | P | 0.364618 | 0.811129223 | 2 |
| GO:0008233 | peptidase activity | F | 0.375375 | 0.004348408 | 2 |
| GO:0016788 | hydrolase activity, acting on ester bonds | F | 0.375375 | 0.41444083 | 1 |
| GO:0050660 | flavin adenine dinucleotide binding | F | 0.431261 | 0.47764437 | 1 |
| GO:0019867 | outer membrane | C | 0.440587 | 0.319024148 | 2 |
| GO:0008237 | metallopeptidase activity | F | 0.453162 | 0.070774432 | 1 |
| GO:0008168 | methyltransferase activity | F | 0.453824 | 0.824820743 | 2 |
| GO:0019538 | protein metabolic process | P | 0.461083 | 8.63E-13 | 4 |
| GO:0050896 | response to stimulus | P | 0.46685 | 0.994848191 | 1 |
| GO:0140096 | catalytic activity, acting on a protein | F | 0.481934 | 0.543682908 | 3 |
